# Implications of the glymphatic system in the diagnostic and surgical workup of normal pressure hydrocephalus

**DOI:** 10.1007/s10072-025-08308-2

**Published:** 2025-06-16

**Authors:** Morgan Broggi, Edoardo M. Barbieri, Alessandro Gans, Pascuzzo Riccardo, Veronica Redaelli, Domenico Aquino, Francesco Restelli, Marco Schiariti, Francesco Acerbi, Giuseppe Di Fede, Paolo Ferroli, Marina Grisoli, Fabio Doniselli

**Affiliations:** 1 Department of Neurosurgery, Fondazione IRCCS Istituto Neurologico Carlo Besta, Via G. Celoria 11, Milano, 20133 Italy; 2 Department of Neurology, Fondazione IRCCS Istituto Neurologico Carlo Besta, Milano, Italy; 3 Department of Neuroradiology, Fondazione IRCCS Istituto Neurologico Carlo Besta, Milano, Italy; 4 Neurosurgery Unit, Pisa University Hospital, Pisa, Italy; 5 Department of Translational Research and New Technology in Medine and Surgery, University of Pisa, Pisa, Italy

**Keywords:** Normal pressure hydrocephalus, Glymphatic System, DTI-ALPS MRI, ALPS-index

## Abstract

**Background:**

Magnetic Resonance (MRI) Diffusion Tensor Imaging Analysis ALong the Perivascular Space (DTI-ALPS) is a promising technique that assesses the glymphatic system (GS) function in many neurodegenerative diseases. This study aims at evaluating the role of DTI-ALPS in the diagnostic and therapeutic management of normal pressure hydrocephalus (NPH).

**Methods:**

Twenty-one NPH patients underwent 3 Tesla MRI DTI-ALPS before and after lumbar tap test (TT). Depending on the response to TT, patients were divided into a responsive cohort (15 responders, R) and non-responsive cohort (6 non-responders, NR). R patients underwent ventriculoperitoneal shunt (VPS) surgery, with clinical assessment upon discharge and at a 3-month follow-up (FU) visit; nine patients repeated DTI-ALPS MRI at FU. Besides, 8 matched healthy controls (HC) underwent the same MRI protocol.

**Results:**

The pre-TT ALPS-index in NPH patients (R: 1.003 ± 0.108, NR: 0.960 ± 0.079) was significantly lower compared to the HC (1.263 ± 0.161, p < 0.01). The pre-TT ALPS-index in R patients was higher than in NR patients, though not significantly (p = 0.39). Compared to the pre-TT values, the ALPS-index of the R group increased both post TT (1.069 ± 0.122, p = 0.0499) and post VPS (1.120 ± 0.117, p = 0.041), in accordance to the clinical outcome.

**Conclusions:**

DTI-ALPS, reflecting the GS function, resulted significantly lower in NPH patients than in healthy controls. Secondly, clinical improvement was associated with DTI-ALPS increase both after a positive response to TT and at long term follow-up following VPS surgery. Therefore, DTI-ALPS index could be a promising, rapid and non-invasive radiological biomarker for the pre-surgical evaluation and prognosis of NPH patients.

## Introduction

Chronic communicating hydrocephalus in adults represents a heterogeneous pathological spectrum, including so-called idiopathic normal pressure hydrocephalus (NPH). NPH is a frequently undiagnosed and misrecognized neurodegenerative disorder, reportedly affecting more than 5% of individuals over the age of 80 [1]. First described in 1965, this condition is a subtype of dementia typically characterized by a triad of symptoms: gait apraxia, urinary incontinence, and cognitive decline [2]. There is also a high incidence of associated parkinsonism [3].

The etiopathogenesis of NPH remains a topic of ongoing debate. Current hypotheses suggest that NPH is an interstitial fluidopathy of the central nervous system (CNS), with dysfunction of the glymphatic system (GS) contributing to its pathophysiology [1, 4]. The GS forms an extramural paravascular network that is closely interconnected with the extracranial lymphatic system. This network facilitates cerebrospinal fluid (CSF) flow dynamics and the removal of extra-vascular hydrophilic metabolic waste and toxic protein aggregates from the brain interstitium. GS dysfunction is associated with progressive interstitial build-up of toxic protein aggregates, such as β-Amyloid and Tau, linked to neurodegeneration and neuroinflammation [5–7]. Several Magnetic Resonance Imaging (MRI) studies, including CNS contrast-enhanced techniques and brain diffusion tensor imaging (DTI) Analysis ALong the Perivascular Space (DTI-ALPS), have demonstrated GS impairment in NPH patients, through a specific DTI-ALPS index [8–14]. The technique provides a reproducible, rapid, and non-invasive method to indirectly infer feasible GS dysfunction [15]. The ALPS-index is usually reduced in NPH and it also correlates with clinical parameters [11–14]. However, its potential to predict surgery outcomes remains largely unexplored.

Ventriculoperitoneal shunt (VPS) surgery is the treatment of choice for NPH [16]; however, selecting suitable surgical candidates remains controversial, since the diagnosis is probabilistic, based on guidelines that do not predict surgical response [17, 18].

Several diagnostic tests are known, including the lumbar tap test (TT), which has sufficiently high positive predictive values to predict surgical response. However, a negative TT result does not necessarily rule out post-surgical improvement, particularly in patients with coexisting parkinsonism [19–21]. Similarly, radiological markers such as Evans index (EI), callosal angle (CA), and disproportionately enlarged subarachnoid space (DESH) provide important diagnostic morphological features, but they all lack sufficient predictive value [22]. Advanced scores and new imaging techniques have been proposed, but their utility remains inconclusive and thus not yet applicable in clinical practice [23–25].

Given the importance of correctly selecting surgical candidates, there is the need for developing suitable non-invasive and replicable predictive parameters.

Herein, we describe the results of a NPH patient cohort undergoing TT, VPS, and DTI-ALPS imaging, focusing primarily on DTI-ALPS index’ role in predicting positive response to TT and to surgery, which was never investigated before. Besides, we speculated on increased GS dysfunction among NPH patients unresponsive to TT, and we explored the association between perivascular diffusivity, CSF neurodegeneration markers, and clinical findings at different time points, before and after TT and after VPS.

## Methods

### Design and timepoints

This study was conducted in accordance with the Declaration of Helsinki principles and was approved by the Ethics Committee (Ref. No. 83/2021, April 14th, 2021) of the Fondazione IRCCS Istituto Neurologico Carlo Besta, Milano, Italy.

Patients attending the NPH outpatient clinic were prospectively enrolled. The inclusion criteria were: (i) clinical and radiological diagnosis of probable NPH according to existing guidelines and (ii) age of over 50 years [16]. Exclusion criteria were: (i) inability to undergo MRI; (ii) a diagnosis of any other neurological pathology, particularly neurodegenerative or neuroinflammatory; (iii) any previous neurosurgical interventions on the brain; (iv) inability to undergo general anaesthesia (GA).

Enrolled patients followed a diagnostic-therapeutic pathway, consisting of the following:

- t_0_: MRI with DTI-ALPS index calculation;
- t_1_ (within 7 days from T_0_): clinical and neurosurgical assessment with NPH-specific rating scales (detailed below) before and after a lumbar TT, including measurements of opening pressure, drainage of 40–50 mL of CSF with dosage of neurodegenerative biomarkers (Aβ-Amyloid 40 and 42; total and phosphorilated (p-)tau). An MRI with DTI-ALPS study within 6 h from TT;
- t_2_ (within 3 months from T_1_): patients with a positive TT response (responders group, R) underwent VPS surgery, with CSF dosage of the same neurodegenerative biomarkers;
- t_3_ (three months after surgery, R group only): clinical and neurosurgical evaluation using NPH-specific rating scales to classify patients as improved, unchanged, or worsened compared to their preoperative condition; MRI with DTI-ALPS study.

Patients unresponsive to TT (non-responders group, NR) were only assessed at t_0_ and t_1_ timepoints.

A healthy control group of patients (HC group) matched for age with the R group, underwent MRI with DTI-ALPS study. Exclusion criteria for this group were: (i) a previous diagnosis of any neurological disease. (ii) a history of any neurosurgical intervention; and (iii) a prior diagnosis of malignancy of any type.

### NPH-specific rating scales

At the t_1_ evaluation stage, a comprehensive neurological assessment was conducted, including computation of the Japanese Normal Pressure Hydrocephalus Grading Score Revised (JNPHGSR), Tinetti Score, Timed Up and Go Test (TUG), Mini-Mental State Examination (MMSE), and Frontal Assessment Battery (FAB) [26–29], all conducted before the TT. Following it, the Tinetti and TUG scores were reassessed.

Patients demonstrating subjective and objective clinical improvement based on the scores after the TT were scheduled for VPS surgery. A three-month follow-up (FU) visit was conducted in an outpatient setting, during which the patient’s clinical status was re-evaluated by assessing the JNPHGSR score, Tinetti Score, TUG times, and radiological status.

### MR study characteristics and DTI-ALPS analysis

Morphological radiological indicators known to be associated with the diagnosis of NPH were measured by an experienced neuroradiologist (> 10 years): these included the EI, CA, NPH Radscale and presence of DESH [30].

**Fig. 1:**
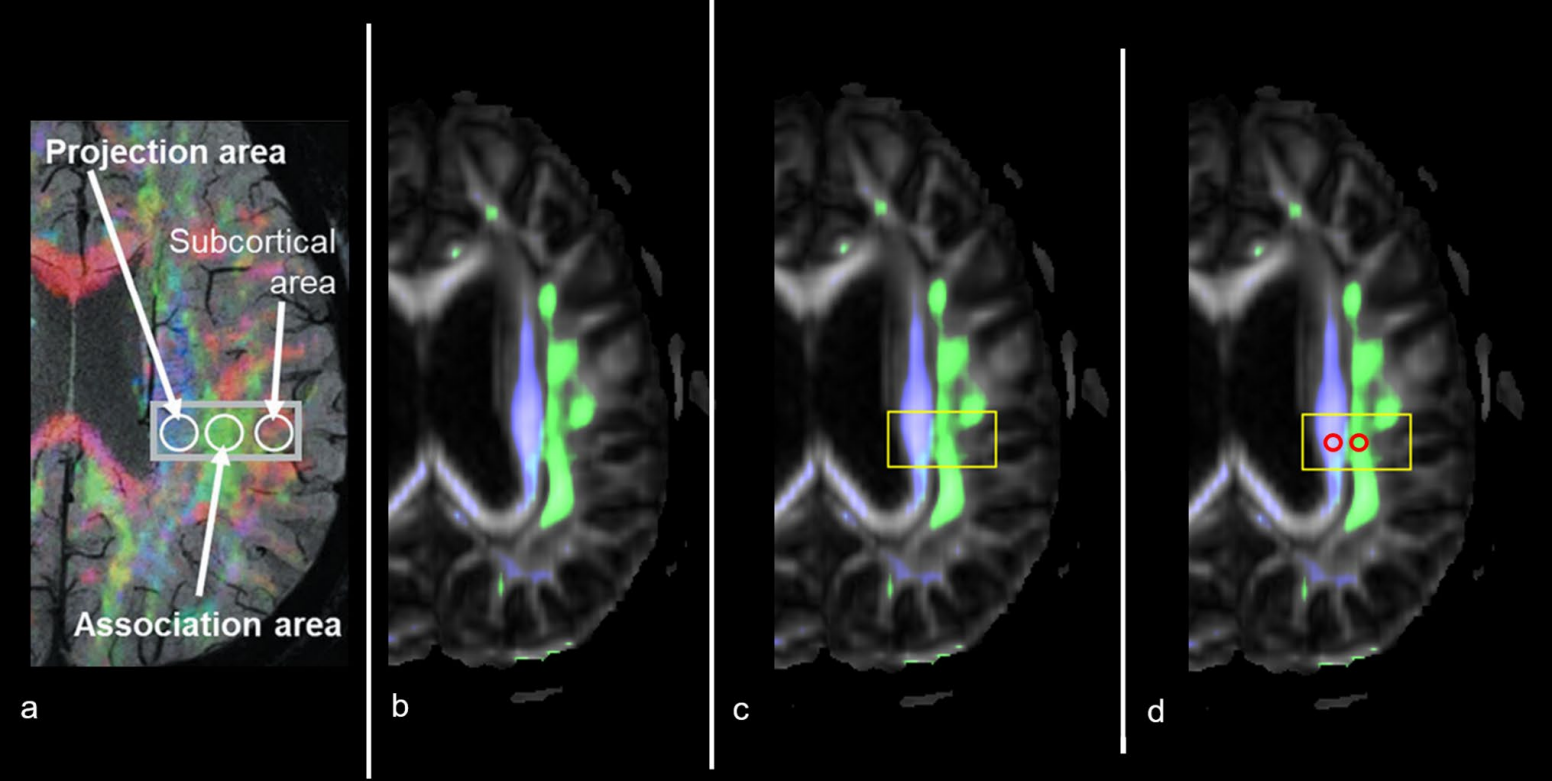
Semi-automatic estimation of the diffusion tensor imaging (DTI) Analysis ALong the Perivascular Space (DTI-ALPS) index was performed by thresholding the fractional anisotropy (FA) color map to isolate projection and association fibers, followed by identification of peak FA voxels within manually defined rectangular Regions of Interest (ROIs) in the periventricular white matter. **a** Adapted from Taoka et al.,[31]; **b** original fractional anisotropy map with overlaid projection (blue) and association (green) fiber areas; **c** manually defined rectangular ROI; **d** two circular ROIs (5 mm) were automatically placed in regions of highest fractional anisotropy

Images were acquired using a 3 T MR Scanner (Achieva dSTREAM, Philips Healthcare, Best, Netherlands). The protocol includes a multi-shell dMRI-ALPS sequence, as mentioned already described in the literature, with the following parameters [31]: voxel size of 2.5 × 2.5x2.5 mm, TR of 8400 ms, TE of 85 ms, flip angle of 90°, slices = 60, shell1 with 30 directions, shell2 with 61 directions, and b values of shell1 = 711 s/mm^2^ and shell2 = 2400 s/mm^2^, with the phase direction being PA (Fig. 1a).

The positioning of the valve system and shunt implant was consistently on the right, with intracranial access from the iuxtacoronary hole and reservoir in the parieto-occipital region (see below). To mitigate artefacts in the DTI sequences acquired for ALPS index measurement, only the left hemilateral values were used for all time points.

Diffusion-MR images underwent initial processing to correct for patient motion, signal drifts, geometrical, and eddy current distortions. In a subsequent step, they were co-registered to the structural volume. From the corrected and co-registered image, diffusion tensors were estimated and maps of fractional anisotropy (FA), color FA, and mean diffusivity were calculated. The preprocessing was conducted using FSL (https://fsl.fmrib.ox.ac.uk/fsl/fslwiki).

To avoid radiologist variability in selecting Regions of Interest (ROI), we calculate the ALPS index semi-automatically, with the following steps: i) conversion and thresholding of the FA color map to a green–blue map to isolate the projection and association components of white matter fibers (Fig. 1b). The threshold was determined as the 95th percentile of the FA values for each component. ii) the neuroradiologist delineated two rectangular ROIs (on the left hemisphere) including projecting fibers in the posterior periventricular white matter, to delimitate the areas for ROI searching (Fig. 1c). iii) Identification of points with maximum FA values for the blue and green channels within the selected rectangular region, resulting in two points for patient. iv) Placement of two circular ROIs (5 mm diameter, Fig. 1d) at the selected points, from which the mean Dxprojection, Dxassoc, Dyprojection, and Dzassociation were extracted to estimate left ALPS index, as described in previous reports​​ [31]. These steps were implemented in a single script written in MATLAB (www.mathworks.com).

### VPS procedure and postoperative management

The VPS procedure was performed using a standardized technique to ensure homogeneity within the study population.

Briefly, the procedure is typically conducted as follows: under GA, a right precoronal burr hole is made and a ventricular catheter is positioned in the right frontal horn using a free-hand technique. Once the ventricle is accessed, 2 ml of CSF are collected for the measurement of neurodegenerative biomarkers. The ventricular catheter is then connected to an adjustable valve (either the Miethke ProGAV 2.0 with an antisiphon device, Potsdam, Germany or the Certas programmable valve, Integra Lifesciences, Princeton, New Jersey, USA) and to a distal catheter, which is introduced into the peritoneum running subcutaneously. The valve is initially adjusted based on the opening pressure measurement during the TT.

### NPH-degenerative biomarkers

CSF levels of Aβ40, Aβ42, Aβ42/Aβ40 ratio, total tau, and p-tau were assessed at t_1,_ t_2_ and t_3_, for R group only; besides, for NR group, CFS was collected and analyzed at t_1_ only. Range values were assessed based on those suggested by Fujirebio S.r.l. for the LUMIPULSE G system; established cut-off values for Alzheimer disease (AD) using the same system were also adopted [32–34].

### Statistical analysis

Continuous variables were tested for normal distribution by Shapiro–Wilk test. Mean and standard deviation were reported for normally distributed variables, while in case of non-normal distribution, median and interquartile range (IQR) were used. Between-group comparisons were performed using Pearson’s chi-squared (χ^2^) test or Fisher’s exact test for categorical variables and Mann–Whitney’s U test or unpaired t-test for continuous variables, as appropriate. Within-group comparisons of continuous variables were performed using Wilcoxon signed-rank test or paired t-test, as appropriate. Adjustment for multiple comparisons was performed by controlling the false discovery rate (FDR) with the Benjamini–Hochberg method. Correlations between continuous variables were tested with Spearman’s correlation coefficient. Statistical significance was set at P-value < 0.05.

Statistical analyses were performed using Stata/SE 18.0 (StataCorp LLC, TX, USA) and R 4.3.1 (R Core Team 2023, Vienna, Austria) software.

## Results

### Baseline demographic, clinical and radiological data

**Fig. 2:**
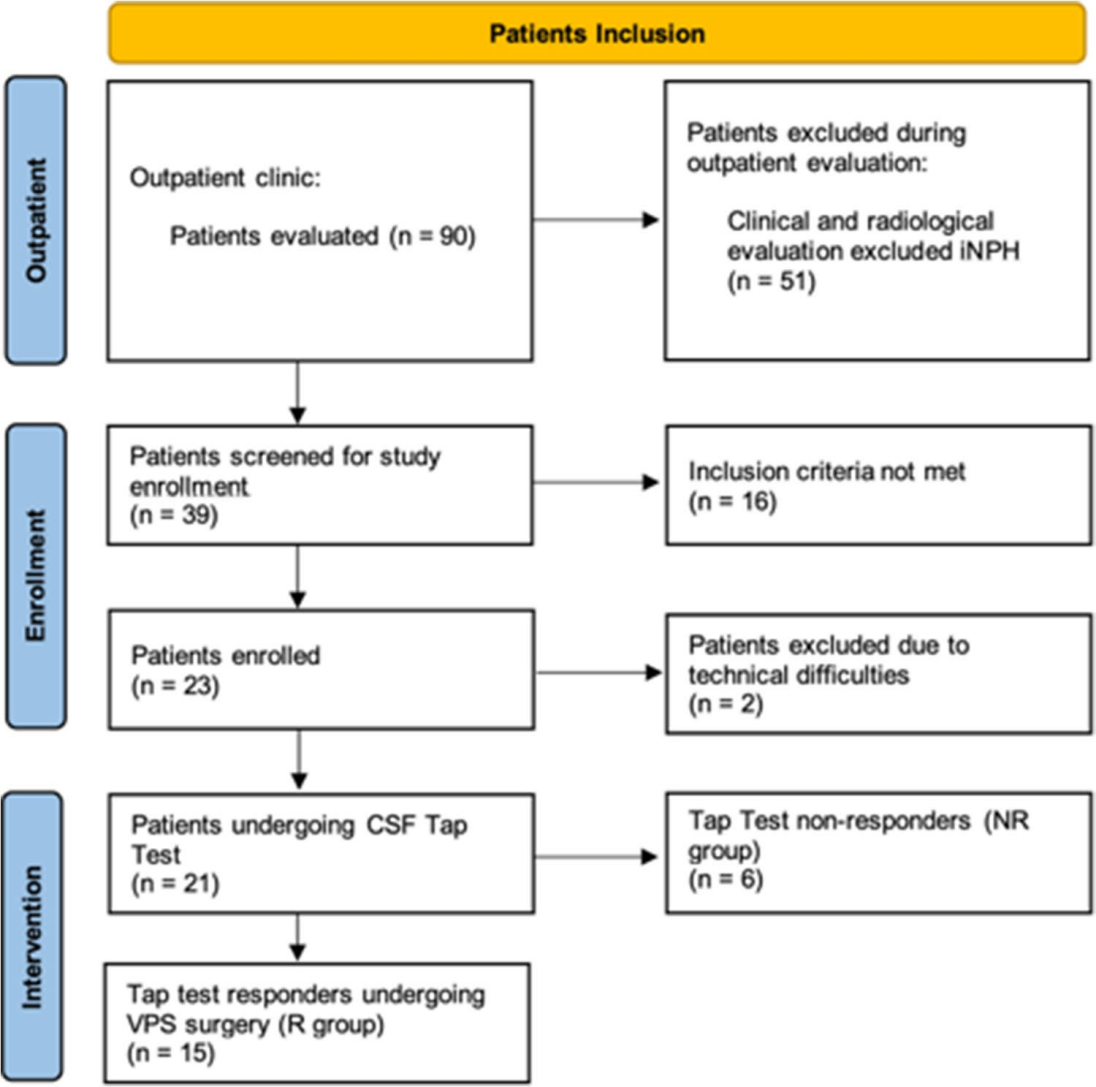
Inclusion flowchart

Between January 2022 and December 2023, 90 patients with probable NPH were evaluated at the outpatient clinic of the Fondazione IRCCS Istituto Neurologico Carlo Besta, Milano, Italy. In 51 cases, the diagnosis of NPH was not confirmed. After applying the aforementioned exclusion criteria, 23 patients (23/39: 59%) were enrolled in the study. Two more patients were then excluded due to technical issues with the MRI, leaving a total of 21 patients (median age 78 years, interquartile range [IQR] 75–81). All patients presented with some degree of gait impairment, but none were completely unable to walk (i.e. JNPHGSR 4 points in gait); 12/21 (57%) had slight/moderate cognitive decline, while 10/21 (48%) complained of urinary disturbances. Six patients (6/21; 28.5%) did not show improvement following the TT and were then excluded from surgery (NR group, median age 80.5 years [IQR 79–82]). Of the 15 TT-responders (R group; 15/21, 71.5%; median age 77 years [IQR 75–80]), nine (9/15; 60%) also underwent DTI-ALPS MRI at the three months FU (t_3_). Demographic and clinical data are summarized in Table 1. The flowchart of the included patients is illustrated in Fig. 2. Eight age-matched HCs were also included.

**Table 1:** Baseline population characteristics

	Enrolled patients (total)		TT-Responders		TT-Non-responders			
Number of patients	21		15		6			
Males (%)	15 (71%)		10 (67%)		5 (83%)			
Females (%)	6 (29%)		5 (33%)		1 (17%)			
	**Median**	**IQR**	**Median**	**IQR**	**Median**		**IQR**	**p-value**
**Clinical parameters**								
Age (years)	78	75—81	77	75–80	80.5	79–82		0.03^*^
JNPHGSR (t_1_ pre-TT)	6	4—7	5	4–7	7	7–8		0.01^*^
MMSE (t_1_)	26	20—29	28	22–30	20.5	20–24		0.08
Tinetti score (t_1_ pre-TT)	17	15—21	18	15.5–22.5	14	10–15		0.02^*^
Tinetti score (t_1_ post-TT)	22	18—24	22	18–24	14	10—17		0.002*
FAB (t_1_)	12	9—15	14	12–16	9.5	8–12		0.08
TUG (s) (t_1_ pre-TT)	24.5	19.5—29.5	22.5	19—27	28.5	26–30		0.22
TUG (s) (t_1_ post-TT)	19.5	16—24.5	18	15–20	26	25—27		0.007*
**Radiological characteristics (t**_**0**_**)**								
Evans Index	0.35	0.33—0.37	0.33	0.33–0.37	0.37	0.35–0.4		0.14
NPH Radscale	8	7—9	8	7–9	7.5	6–9		0.69
Transcallosal angle (°)	80	65—85	80	74—85	82.5	65–90		0.69
**CSF Biomarkers characteristics (t**_**1**_**)**								
Tau protein (pg/ml)	223	159—316	200	150–285	306	207–431		0.11
p-181 (pg/ml)	28.5	21.1—42.2	26.6	20.7–40.6	34.7	26.5–51.4		0.24
Aβ−42 (pg/ml)	496	427—753	463	423–755	620	494–750		0.38
Aβ−40 (pg/ml)	6672	4696—8089	6392.5	4158–8131	7086	5345–7645		0.40
Aβ−42/Aβ−40 ratio	0.10	0.07—0.11	0.101	0.07–0.105	0.096	0.07–0.11		0.93

*CSF* cerebrospinal fluid

*FAB* frontal assessment battery

*JNPHGSR *Japanese normal pressure hydrocephalus grading score revised

*MMSE* mini-mental state examination

*NPH* normal pressure hydrocephalus

*TT* tap test

*TUG* timed up and go test

Male/female ratio was 10/5 (67/33%) for R, 5/1 (83/17%) for NR and 3/5 (38/62%) for HC groups.

Considering separately R and NR groups (Table 1), R were significantly slightly younger (77 vs. 80.5; p = 0.03), with lower baseline JNPHGSR scores (5 vs. 7; p = 0.01) and higher Tinetti scores (18 vs. 14; p = 0.02). The remaining evaluated neurological scales were not significantly different between the two groups.

Concerning radiological features, the median EI was 0.33 (0.33–0.37) for R and 0.37 (0.35–0.40) for NR; median CA was 88° (74°−85°) for R and 82.5° (65°−90°) for NR; median NPH Radscale was 8 (7–9) for R and 7.5 (6–9) for NR. All the radiological features did not significantly differ between the two groups (p = 0.14, p = 0.69, p = 0.69 respectively). DESH presence was detected in all 21 patients.

At t_1_, all analyzed CSF biomarkers did not show any noticeable difference between R and NR groups.

Rather expectedly, after TT we found significantly lower TUG times (18 s vs. 26 s, p = 0.007) and higher Tinetti test scores (23 vs. 14, p = 0.002) in responders than in non-responders. A summary of baseline and post-TT clinical outcomes is reported in Table 1.

### Clinical outcomes

No intraoperative nor early postoperative complications requiring invasive treatment occurred; there was no surgical-related mortality nor morbidity. Two out of 15 R patients (13%) experienced CSF overdrainage, with subdural collections that required valve adjustments only.

All responders showed clinical improvement (i.e. improvement of at least 1 point in the JNPHGSR) after surgery, both early postoperatively and at the 3-month FU. Specifically, at the 3-month FU, 12/15 (80%) patients reported an improved gait, while 3/15 (20%) remained unchanged. Five out of 6 subjects with preoperative cognitive decline, including 2 that did not experience any gait improvement, had higher postoperative MMSE scores, with 1 reporting no change in cognitive function (but an ameliorated gait). Finally, 4/6 (67%) patients with preoperative urinary disturbances, including the third patient without an improvement in gait, reported an at least 1 point improvement in the urinary section of the JNPHGSR; the remaining 2 subjects that complained the same urinary status as the preoperative condition experienced anyway an improvement in balance.

Considering clinical scales, at late FU, TUG times decreased from a preoperative median of 22.5 s (19–27) to 18 (14–24.5, p = 0.005). Median preoperative Tinetti scores (17, 15–21) significantly improved (26.5, 26–27; p = 0.011). Likewise, JNPHGSR scores improved from a median value of 6 (4–7) to 3 (2–4; p = 0.039). The MMSE raised from a median of 28 to 29 (26–30; p = 0.036). Clinical outcomes following VPS in responders are summarized in Table 2.

**Table 2:** Responders (R) group clinical outcome

	Pre-TT		Post-TT			3 months post-VPS		
Clinical Outcomes	Median	IQR	Median	IQR	p-value	Median	IQR	p-value
***n* = 15**			***n* = 15**			***n* = 10**		
TUG	22.5	19–27	19.5	16–24.5	< 0.000*	18	14–24.5	0.005*
Tinetti	17	15–21	22	18–24	0.002^*^	26.5	26–27	0.011^*^
MMSE	28	22—30	-	-	-	29	26—30	0.036^*^
JNPHGSR	6	4–7	-	-	-	3	2–4	0.039^*^

*JNPHGSR* Japanese normal pressure hydrocephalus grading score revised

*TT* tap test

*TUG* timed up and go test

*VPS* ventriculoperitoneal shunt

### CSF neurogenerative biomarkers (R group only)

The concentration of CSF biomarkers varied significantly during the process. More specifically, median total Tau protein concentration increased from 200 pg/ml (150–285) upon TT (t_1_) to 1278 pg/ml (851–2000; p = 0.01) during surgery (t_2_) to 455 pg/ml (258–614; p = 0.02) at follow-up (t_3_). Similarly, median p-tau values increased from 26.6 pg/ml (20.7–40.6) to 88.5 pg/ml (60.5–239; p = 0.01) and 59.7 (33–79; p = 0.01), respectively. Likewise, median Aβ40 concentration increased from 6392.5 pg/ml (4158–8131) at (t_1_) to 9294 pg/ml (6424–11876; p = 0.04) at (t_3_); interestingly, median Aβ40 values were significantly lower during surgery (4955, 4014–5918; p = 0.04). All biomarkers-related outcomes are summarized in detail in Table 3.

**Table 3:** Cerebrospinal fluid (CSF) Biomarkers dosage in R group

	Tap Test		VPS			3 months post-VPS		
**C**linical Outcomes	Median	IQR	Median	IQR	p-value	Median	IQR	p-value
***n* = 15**			***n* = 15**			***n* = 10**		
Total Tau (pg/ml)	200	150–285	1278	851—2000	0.01^*^	455	258—614	0.02^*^
p-tau (pg/ml)	26.6	20.7–40.6	88.5	60.5–239	0.01^*^	59.7	33–79	0.01^*^
Aβ−42 (pg/ml)	463	423–755	475	382—588	0.06	730	439—1232	0.06
Aβ−40 (pg/ml)	6392.5	4158–8131	4955	4014—5918	0.04^*^	9294	6424—11,876	0.04^*^
Aβ−42/Aβ−40	0.101	0.07–0.105	0.1	0.1–0.1	0.7	0.09	0.09–0.1	0.3

*IQR* interquartile range

*VPS* ventriculoperitoneal shunt

### DTI-ALPS values

The pre-TT ALPS index of patients with NPH (either R [1.003 ± 0.108] or NR [0.960 ± 0.079]) was significantly lower compared to the HC group (1.263 ± 0.161; FDR-adjusted p = 0.0004 and 0.0018, respectively) (Table 4). The pre-TT ALPS index of responders was higher compared to non-responders, but this did not reach significance (p = 0.3852).

**Table 4:** Left DTI-ALPS index values by group over time

Time	Left DTI-ALPS index, mean (SD)		
	*Responder*	*Non-responder*	*HC*
*T0 – baseline*	1.003 (0.108) ^†^	0.960 (0.079) ^§^	1.263 (0.161)
*T1 – post-TT*	1.069 (0.122)	0.991 (0.078)	NA
*T3 – post-VPS*	1.120 (0.117)	NA	NA

^†^ Significantly lower than HC, FDR-adjusted *p* = 0.0004

^§^ Significantly lower than HC, FDR-adjusted *p* = 0.0018

*DTI-ALPS* diffusion tensor imaging analysis along the perivascular space

*FDR* false discovery rate

*HC* healthy control

*NA* not available

*SD* standard deviation

*TT* tap test

*VPS* ventriculoperitoneal shunt

**Fig. 3:**
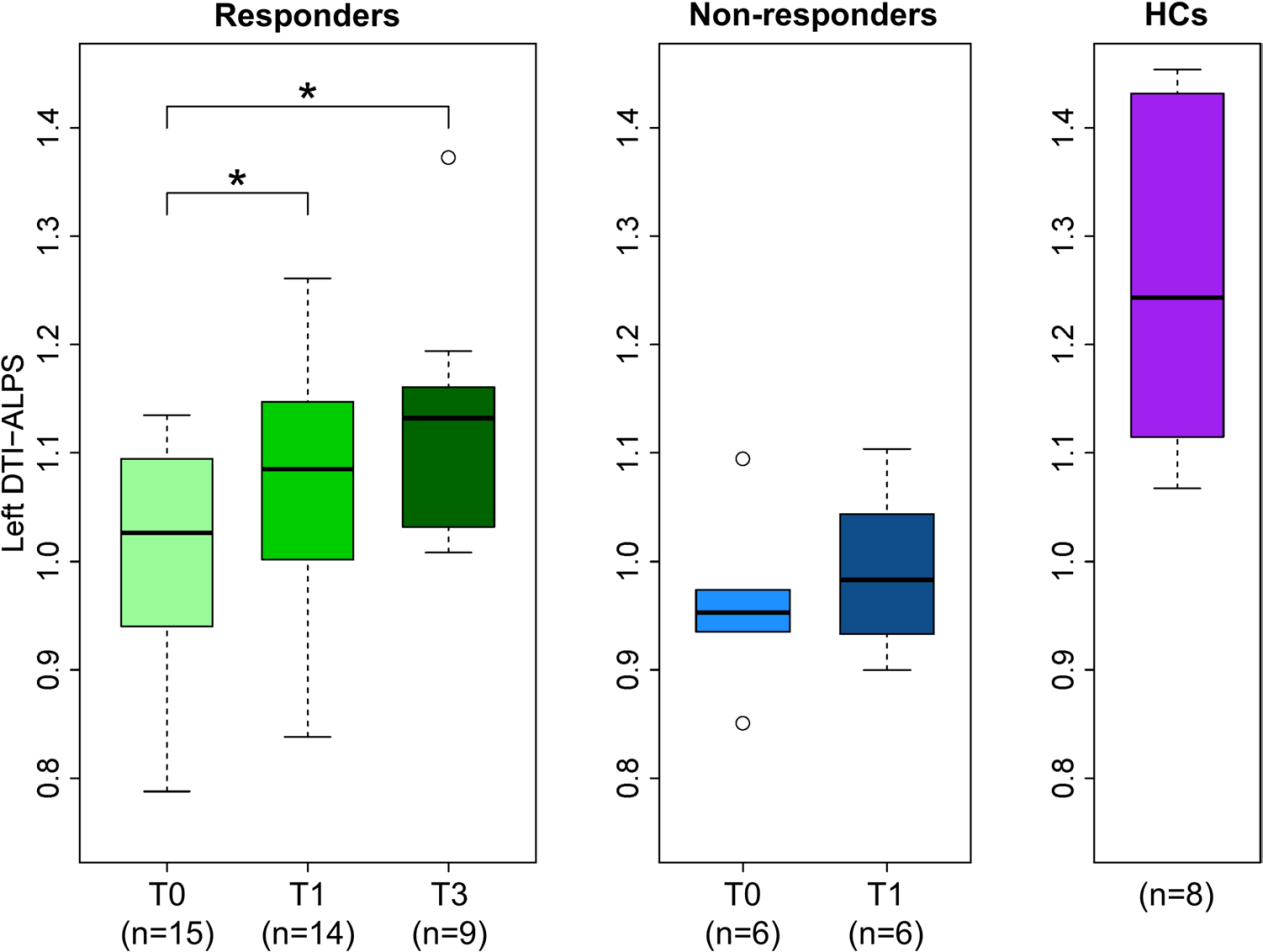
Boxplots showing the distribution of diffusion tensor imaging (DTI) Analysis ALong the Perivascular Space (DTI-ALPS) values within groups and across timepoints. Asterisks denote significantly different DTI-ALPS values between timepoints. HCs = healthy controls; T0 = pre tap test; T1 = post tap test; T3 = post ventriculoperitoneal shunt

The ALPS index of responders showed a significant increase between pre-TT and post-TT (1.003 ± 0.108 vs 1.069 ± 0.122, FDR-adjusted p = 0.0499), as well as a significant increase between pre-TT and post-VPS (1.003 ± 0.108 vs 1.120 ± 0.117, FDR-adjusted p = 0.0408), as illustrated in Fig. 3. In contrast, pre- and post-TT ALPS indices were not significantly different in non-responders (0.960 ± 0.079 vs 0.991 ± 0.078, FDR-adjusted p = 0.3630).

DTI-ALPS index did not significantly correlate with EI (ρ = −0.328, p = 0.147), CA (ρ = −0–117, p = 0.632) and Radscale (ρ = 0.109, p = 0.639).

## Discussion

We analyzed the brain hydrodynamics of patients affected by NPH via DTI-ALPS index, a non-invasive radiological biomarker for studying the human GS in vivo [15]. To our knowledge, this is the first study that assesses the variation of DTI-ALPS index following both TT and VPS in patients affected by NPH. Our results demonstrated that NPH patients have a significantly lower mean DTI-ALPS index than HC, confirming the hypothesis that a GS dysfunction is involved in NPH pathogenesis.

Moreover, TT-responders had a higher pre-procedural mean DTI-ALPS index compared to non-responders, although not significant. Immediately after TT, responders experienced a significant increase in the index, that was maintained 3 months after VPS. The clinical significance of DTI-ALPS index was further strengthened by the finding that non-responders showed no change before and after TT.

Previous studies showed that DTI-ALPS index is significantly lower in patients with NPH compared to pseudo-NPH cases and healthy subjects and our data confirm these findings [11, 12]. Interestingly, a study of 16 patients with NPH found significantly higher DTI-ALPS index values in subjects responsive to the TT compared to non-responsive ones [12]. In contrast, our study did not reveal a significant difference between the two groups, although the mean DTI-ALPS index values at t_0_ were higher in responders.

This suggests that the DTI-ALPS index may be helpful in predicting both TT and VPS response and could be included in the preoperative workup to aid in patient selection.

In a study of 9 NPH patients undergoing lumboperitoneal shunt, the DTI-ALPS index varied significantly between pre- and post-operative measurements for the entire cohort and in responsive patients when analyzed separately. In contrast, no significant changes were observed in non-responders [13].

In our analysis, the DTI-ALPS index increased significantly not only between pre- and post-TT in responders, but also post-operatively at three months. This may indicate a long-term GS recovery, in accordance with clinical improvement in these patients.

To date, despite contradictory reports in the literature, the only accepted radiological predictors of shunt responsiveness are the CA and periventricular white matter alterations [22, 35, 36]. In our study, all patients presented with the diagnostic radiological parameters indicated in international guidelines. Considering the entire cohort of patients, the Evans Index (0.36; IQR 0.04) and the CA (80; IQR 20) were always within the threshold values defined for NPH diagnosis [36].

The EI and CA considered in our cohort of responsive and non-responsive patients were not significantly different, thus lacking predictive value for the response to VPS. Moreover, in contrast to previous evidence, we did not find a significant correlation between these radiological parameters and DTI-ALPS index [12, 13].

Concerning clinical NPH features, gait apraxia was present in at least 90% of cases according to the current literature, followed by urinary disturbances and cognitive decline, which are variably present at clinical presentation but are both observed in 80% of patients during the natural course of the disease [18, 37, 38]. Baseline MMSE and FAB revealed a trend towards higher values in R group compared NR, although not statistically significant. This finding suggests a higher likelihood of responsiveness to CSF drainage in patients with a greater baseline cognitive preservation.

Likewise, patients in our cohort that resulted responsive to TT showed an overall better baseline clinical status, as reflected by lower baseline JNPHGSR scores (p = 0.01), and younger age (p = 0.03) compared to non-responders. This finding simultaneously underlines the importance of early detection of NPH, in order to provide patients with the best chance of clinical improvement with CSF diversion procedures.

All R patients underwent VPS and showed significant clinical improvement at the three-month follow-up, aligning with the 73–96% success rate reported in the literature [39]. Improvement was evaluated both subjectively by patients interviews and objectively through the aforementioned clinical scores.

When we consider CSF biomarkers of neurodegeneration values, we observed that NPH patients showed a pattern characterised by reduction of amyloid proteins, with conservation of the ratio, normal level of total and p-Tau, or even reduced, compared to the normal values. These observations could suggest a dilution of proteins due to the presence of NPH, though this interpretation remains uncertain. Looking at the results obtained from the CSF taken during VPS, we observed a high level of tau proteins, likely due to the mechanical trauma caused by the catheter insertion. Amyloid proteins as well showed pathological increase, but the ratio continues to be normal. Eventually, the restoration of the glymphatic homeostasis would also be evidenced by the increase in neurodegeneration markers collected at follow-up, indicating enhanced clearance of protein aggregates from the interstitium [40], thus slowing the neurodegenerative process. Nevertheless, differences in CSF composition may also be influenced by the different collection modalities and locations, namely lumbar puncture during TT and intraventricular CSF collection during surgery and at follow-up.

These neurodegeneration markers appear to aid in the often complex differential diagnosis of NPH with AD, fronto-temporal dementia, progressive supranuclear palsy and vascular dementia. In NPH patients, β-Amyloid peptides 42 and 40 measured in CSF are significantly reduced compared to the healthy population but are not dissimilar to those in AD patients; however, total Tau and p-tau are significantly lower compared to AD patients [41, 42]. The primary hypotheses regarding the reduction of amyloid peptide levels in NPH patients involve the possibility of a CSF dilution effect and the deposition of interstitial aggregates secondary to GS impairment. Within the NPH population, reduced neurodegeneration marker values are a predictive element of response to CSF drainage [1, 43, 44].

In line with our study's results, a previous work suggested that the increase in CSF levels of neurodegeneration markers in NPH patients is due to the improvement of interstitial drainage post-VPS [45], confirmed by the rise in perivascular diffusivity in DTI-ALPS index and, consequently, the function of the GS.

Although our study sheds further light on the pathophysiology and management of NPH, we are conscious that it presents some limitations. Firstly, the limited number of patients and healthy controls highlights the need for a larger sample size to more reliably assess glymphatic function in NPH patients. Secondly, the DTI-ALPS method is a newly developed technique, still needing more uniform standardization in ROI calculation and placement as well as standardization across different scanners, which still represent a source of great variability. Lastly, although our data suggest a possible role for DTI-ALPS index in predicting shunt responsiveness, we must acknowledge that this should be considered an indirect measure of the GS [31]. Indeed, the observed changes in the ALPS index may not exclusively indicate improved GS function but could instead reflect other processes influenced by CSF drainage and shunt placement, such as altered brain compliance or shifts in interstitial pressure gradients. Therefore, our preliminary findings are not yet robust enough to incorporate this technique in the everyday clinical practice and larger studies are needed to corroborate our hypotheses.

## Conclusions

Despite an increasing prevalence of NPH, the disease diagnostic and therapeutic process still presents substantial challenges. These hurdles can be explained by an incomplete understanding of its pathogenesis. The current, most widely accepted hypothesis points towards a CNS interstitial fluidopathy, characterized by GS dysfunction and consequent impairment of normal neurofluid dynamics. Integrating the GS study into routine clinical practice may hence reveal helpful both in the surgical selection of NPH patients and in estimating the degree of cerebral tissue damage. Among the possible diagnostic methodologies, DTI-ALPS seems a rapid, reproducible, and non-invasive technique. To this regard, our study is pioneer in observing the GS response to TT and VPS. Furthermore, the identified tendency towards higher baseline DTI-ALPS index values in patients selected for surgery, as well as the presence of some degree of postoperative improvement in all of them, indicate a possible future role of DTI-ALPS in the selection process of surgical patients.

## Data Availability

The data presented in this study are available upon request to the corresponding author.

## References

[1] Reeves BC et al (2020) Glymphatic system impairment in Alzheimer’s disease and idiopathic normal pressure Hydrocephalus. Trends Mol Med 26(3):285–295. 10.1016/J.MOLMED.2019.11.008 31959516 10.1016/j.molmed.2019.11.008 PMC7489754

[2] Adams RD, Fisher CM, Hakim S, Ojemann RG, Sweet WH (1965) Symptomatic occult hydrocephalus with normal cerebrospinal-fluid pressure. Va Med Mon (1918) 97(11):693–695. 10.1056/NEJM196507152730301

[3] Mostile G, Fasano A, Zappia M (2022) Parkinsonism in idiopathic normal pressure hydrocephalus: is it time for defining a clinical tetrad? Neurol Sci 43(9):5201–5205. 10.1007/S10072-022-06119-3 35648268 10.1007/s10072-022-06119-3 PMC9385815

[4] Taoka T, Naganawa S (2021) Imaging for central nervous system (CNS) interstitial fluidopathy: disorders with impaired interstitial fluid dynamics. Jpn J Radiol 39(1):1–14. 10.1007/S11604-020-01017-0/TABLES/1 32653987 10.1007/s11604-020-01017-0 PMC7813706

[5] Hladky SB and Barrand MA (2022) The glymphatic hypothesis: the theory and the evidence. Fluids Barriers CNS 19(1) 10.1186/S12987-021-00282-Z 10.1186/s12987-021-00282-z PMC8815211 35115036

[6] Rasmussen MK, Mestre H, Nedergaard M (2022) Fluid transport in the brain. Physiol Rev 102(2):1025–1151. 10.1152/PHYSREV.00031.2020 33949874 10.1152/physrev.00031.2020 PMC8897154

[7] Nedergaard M, Goldman SA (2020) Glymphatic failure as a final common pathway to dementia. Science 370(6512):50. 10.1126/SCIENCE.ABB8739 33004510 10.1126/science.abb8739 PMC8186542

[8] Ringstad G, Vatnehol SAS, Eide PK (2017) Glymphatic MRI in idiopathic normal pressure hydrocephalus. Brain 140(10):2691–2705. 10.1093/BRAIN/AWX191 28969373 10.1093/brain/awx191 PMC5841149

[9] Eide PK, Ringstad G (2019) Delayed clearance of cerebrospinal fluid tracer from entorhinal cortex in idiopathic normal pressure hydrocephalus: a glymphatic magnetic resonance imaging study. J Cereb Blood Flow Metab 39(7):1355–1368. 10.1177/0271678X18760974 29485341 10.1177/0271678X18760974 PMC6668515

[10] Eide PK et al (2022) Intrathecal contrast-enhanced magnetic resonance imaging of cerebrospinal fluid dynamics and glymphatic enhancement in idiopathic normal pressure hydrocephalus. Front Neurol 13.10.3389/FNEUR.2022.857328 10.3389/fneur.2022.857328 PMC9019061 35463139

[11] Yokota H et al (2019) Diagnostic performance of glymphatic system evaluation using diffusion tensor imaging in idiopathic normal pressure hydrocephalus and mimickers. Curr Gerontol Geriatr Res 2019 10.1155/2019/5675014 10.1155/2019/5675014 PMC6609364 31320896

[12] Bae YJ, Choi BS, Kim JM, Choi JH, Cho SJ, Kim JH (2021) Altered glymphatic system in idiopathic normal pressure hydrocephalus. Parkinsonism Relat Disord 82:56–60. 10.1016/J.PARKRELDIS.2020.11.009 33248394 10.1016/j.parkreldis.2020.11.009

[13] Kikuta J et al (2022) Water diffusivity changes along the perivascular space after lumboperitoneal shunt surgery in idiopathic normal pressure hydrocephalus. Front Neurol 13. 10.3389/FNEUR.2022.843883 10.3389/fneur.2022.843883 PMC8918529 35295837

[14] Georgiopoulos C et al (2024) Noninvasive assessment of glymphatic dysfunction in idiopathic normal pressure hydrocephalus with diffusion tensor imaging. J Neurosurg 140(3):612–620. 10.3171/2023.6.JNS23260 37724800 10.3171/2023.6.JNS23260

[15] Taoka T et al (2017) Evaluation of glymphatic system activity with the diffusion MR technique: diffusion tensor image analysis along the perivascular space (DTI-ALPS) in Alzheimer’s disease cases. Jpn J Radiol 35(4):172–178. 10.1007/S11604-017-0617-Z 28197821 10.1007/s11604-017-0617-z

[16] Bergsneider M, Black PM, Klinge P, Marmarou A, Relkin N (2005) Surgical management of idiopathic normal-pressure hydrocephalus. Neurosurgery 57(suppl_3):S2-29-S2-39. 10.1227/01.NEU.0000168186.45363.4D 10.1227/01.neu.0000168186.45363.4d 16160427

[17] Relkin N, Marmarou A, Klinge P, Bergsneider M, McL Black P (2005) Diagnosing idiopathic normal-pressure hydrocephalus. Neurosurgery 57(3 Suppl):S24–S216. 10.1227/01.NEU.0000168185.29659.C5 16160425 10.1227/01.neu.0000168185.29659.c5

[18] Nakajima M et al (2021) Guidelines for management of idiopathic normal pressure hydrocephalus (Third Edition): endorsed by the Japanese society of normal pressure hydrocephalus. Neurol Med Chir (Tokyo) 61(2):63. 10.2176/NMC.ST.2020-0292 33455998 10.2176/nmc.st.2020-0292 PMC7905302

[19] Wikkelsø C, Hellström P, Klinge PM, Tans JTJ (2013) The European iNPH multicentre study on the predictive values of resistance to CSF outflow and the CSF Tap Test in patients with idiopathic normal pressure hydrocephalus. J Neurol Neurosurg Psychiatry 84(5):562–568. 10.1136/JNNP-2012-303314 23250963 10.1136/jnnp-2012-303314

[20] Marmarou A, Bergsneider M, Klinge P, Relkin N and Black PML (2005) The value of supplemental prognostic tests for the preoperative assessment of idiopathic normal-pressure hydrocephalus. Neurosurgery 57(3 Suppl).10.1227/01.NEU.0000168184.01002.60 10.1227/01.neu.0000168184.01002.60 16160426

[21] Giannini G et al (2023) INPH and parkinsonism: a positive shunt response with a negative tap test. Front Neurol 14:1150258. 10.3389/FNEUR.2023.1150258 37064209 10.3389/fneur.2023.1150258 PMC10090367

[22] Carlsen JF, Munch TN, Hansen AE, Hasselbalch SG, Rykkje AM (2022) Can preoperative brain imaging features predict shunt response in idiopathic normal pressure hydrocephalus? A PRISMA review. Neuroradiology 64(11):2119–2133. 10.1007/S00234-022-03021-9 35871239 10.1007/s00234-022-03021-9

[23] Shinoda N et al (2017) Utility of MRI-based disproportionately enlarged subarachnoid space hydrocephalus scoring for predicting prognosis after surgery for idiopathic normal pressure hydrocephalus: clinical research. J Neurosurg 127(6):1436–1442. 10.3171/2016.9.JNS161080 28156249 10.3171/2016.9.JNS161080

[24] Karki P et al (2024) Prediction of surgical outcomes in normal pressure hydrocephalus by MR elastography. AJNR Am J Neuroradiol 45(3):328–334. 10.3174/AJNR.A8108 38272572 10.3174/ajnr.A8108 PMC11286123

[25] Algin O, Hakyemez B, Parlak M (2010) The efficiency of PC-MRI in diagnosis of normal pressure hydrocephalus and prediction of shunt response. Acad Radiol 17(2):181–187. 10.1016/J.ACRA.2009.08.011 19910214 10.1016/j.acra.2009.08.011

[26] Akiguchi I et al (2008) Shunt-responsive parkinsonism and reversible white matter lesions in patients with idiopathic NPH. J Neurol 255(9):1392–1399. 10.1007/s00415-008-0928-1 18575921 10.1007/s00415-008-0928-1

[27] Tinetti ME (1986) Performance-oriented assessment of mobility problems in elderly patients. J Am Geriatr Soc 34(2):119–126. 10.1111/j.1532-5415.1986.tb05480.x 3944402 10.1111/j.1532-5415.1986.tb05480.x

[28] Podsiadlo D, Richardson S (1991) The timed “up & go”: a test of basic functional mobility for frail elderly persons. J Am Geriatr Soc 39(2):142–148. 10.1111/j.1532-5415.1991.tb01616.x 1991946 10.1111/j.1532-5415.1991.tb01616.x

[29] Folstein MF, Folstein SE, McHugh PR (1975) “Mini-mental state.” J Psychiatr Res 12(3):189–198. 10.1016/0022-3956(75)90026-6 1202204 10.1016/0022-3956(75)90026-6

[30] Kockum K et al (2018) The idiopathic normal-pressure hydrocephalus Radscale: a radiological scale for structured evaluation. Eur J Neurol 25(3):569–576. 10.1111/ene.13555 29281156 10.1111/ene.13555

[31] Taoka T, Ito R, Nakamichi R, Nakane T, Kawai H, Naganawa S (2024) Diffusion Tensor Image Analysis ALong the Perivascular Space (DTI-ALPS): Revisiting the Meaning and Significance of the Method. Magn Reson Med Sci 23(3):2023–0175. 10.2463/mrms.rev.2023-0175 10.2463/mrms.rev.2023-0175 PMC11234944 38569866

[32] Blennow K, Zetterberg H (2013) The application of cerebrospinal fluid biomarkers in early diagnosis of Alzheimer disease. Med Clin North Am 97(3):369–376. 10.1016/j.mcna.2012.12.012 23642576 10.1016/j.mcna.2012.12.012

[33] Leitão MJ et al (2019) Clinical validation of the lumipulse G cerebrospinal fluid assays for routine diagnosis of Alzheimer’s disease. Alzheimers Res Ther 11(1):91. 10.1186/s13195-019-0550-8 31759396 10.1186/s13195-019-0550-8 PMC6875031

[34] Bellomo G et al (2021) Machine learning driven profiling of cerebrospinal fluid core biomarkers in Alzheimer’s disease and other neurological disorders. Front Neurosci 15. 10.3389/fnins.2021.647783 10.3389/fnins.2021.647783 PMC8044304 33867925

[35] Chen J et al (2022) Value of MRI-based semi-quantitative structural neuroimaging in predicting the prognosis of patients with idiopathic normal pressure hydrocephalus after shunt surgery. Eur Radiol 32(11):7800–7810. 10.1007/S00330-022-08733-3 35501572 10.1007/s00330-022-08733-3 PMC9668801

[36] Park HY, Kim M, Suh CH, Lee DH, Shim WH, Kim SJ (2021) Diagnostic performance and interobserver agreement of the callosal angle and Evans’ index in idiopathic normal pressure hydrocephalus: a systematic review and meta-analysis. Eur Radiol 31(7):5300–5311. 10.1007/s00330-020-07555-5 33409775 10.1007/s00330-020-07555-5

[37] Williams MA and Malm J (2016) Diagnosis and treatment of idiopathic normal pressure hydrocephalus. Continuum (Minneap Minn) 22(2);579–599. 10.1212/CON.0000000000000305 10.1212/CON.0000000000000305 PMC5390935 27042909

[38] Campos-Juanatey F, Gutiérrez-Baños JL, Portillo-Martín JA, Zubillaga-Guerrero S (2015) Assessment of the urodynamic diagnosis in patients with urinary incontinence associated with normal pressure hydrocephalus. Neurourol Urodyn 34(5):465–468. 10.1002/NAU.22600 24729303 10.1002/nau.22600

[39] Isaacs AM, Williams MA and Hamilton MG (2019) Current update on treatment strategies for idiopathic normal pressure Hydrocephalus. Curr Treat Options Neurol 21(12).10.1007/S11940-019-0604-Z 10.1007/s11940-019-0604-z 31792620

[40] Graff-Radford NR (2014) Alzheimer CSF biomarkers may be misleading in normal-pressure hydrocephalus. Neurology 83(17):1573–1575. 10.1212/WNL.0000000000000916 25332445 10.1212/WNL.0000000000000916 PMC4222848

[41] Santangelo R et al (2017) Cerebrospinal fluid Amyloid-β 42, total tau and phosphorylated tau are low in patients with normal pressure hydrocephalus: analogies and differences with Alzheimer’s disease. J Alzheimer’s Dis 60(1):183–200. 10.3233/JAD-170186 28826180 10.3233/JAD-170186

[42] Kapaki EN et al (2007) Cerebrospinal fluid tau, phospho-tau tau 181 and β ‐amyloid 1−42 in idiopathic normal pressure hydrocephalus: a discrimination from Alzheimer’s disease. Eur J Neurol 14(2):168–173. 10.1111/j.1468-1331.2006.01593.x 17250725 10.1111/j.1468-1331.2006.01593.x

[43] Santangelo R et al (2017) Cerebrospinal fluid amyloid-β 42, total tau and phosphorylated tau are low in patients with normal pressure hydrocephalus: analogies and differences with Alzheimer’s disease. J Alzheimers Dis 60(1):183–200. 10.3233/JAD-170186 28826180 10.3233/JAD-170186

[44] Darrow JA et al (2022) CSF Biomarkers predict gait outcomes in idiopathic normal pressure hydrocephalus. Neurol Clin Pract 12(2):91–101. 10.1212/CPJ.0000000000001156 35733946 10.1212/CPJ.0000000000001156 PMC9208405

[45] Lidén S, Farahmand D, Laurell K (2022) Ventricular volume in relation to lumbar CSF levels of amyloid-β 1–42, tau and phosphorylated tau in iNPH, is there a dilution effect? Fluids Barriers CNS 19(1):1–9. 10.1186/S12987-022-00353-9/TABLES/2 35843939 10.1186/s12987-022-00353-9 PMC9288679

